# Nothing in Evolution Makes Sense Except in the Light of Genomics: Read–Write Genome Evolution as an Active Biological Process

**DOI:** 10.3390/biology5020027

**Published:** 2016-06-08

**Authors:** James A. Shapiro

**Affiliations:** Department of Biochemistry and Molecular Biology, University of Chicago, GCIS W123B, 979 E. 57th Street, Chicago, IL 60637, USA; jsha@uchicago.edu; Tel.: +1-773-702-1625; Fax: +1-773-947-9345

**Keywords:** genome sequence, molecular phylogeny, repetitive DNA, symbiogenesis, hybrid speciation, whole genome duplication, horizontal DNA transfer, viviparity, mobile DNA elements, stem cell pluripotency

## Abstract

The 21st century genomics-based analysis of evolutionary variation reveals a number of novel features impossible to predict when Dobzhansky and other evolutionary biologists formulated the neo-Darwinian Modern Synthesis in the middle of the last century. These include three distinct realms of cell evolution; symbiogenetic fusions forming eukaryotic cells with multiple genome compartments; horizontal organelle, virus and DNA transfers; functional organization of proteins as systems of interacting domains subject to rapid evolution by exon shuffling and exonization; distributed genome networks integrated by mobile repetitive regulatory signals; and regulation of multicellular development by non-coding lncRNAs containing repetitive sequence components. Rather than single gene traits, all phenotypes involve coordinated activity by multiple interacting cell molecules. Genomes contain abundant and functional repetitive components in addition to the unique coding sequences envisaged in the early days of molecular biology. Combinatorial coding, plus the biochemical abilities cells possess to rearrange DNA molecules, constitute a powerful toolbox for adaptive genome rewriting. That is, cells possess “Read–Write Genomes” they alter by numerous biochemical processes capable of rapidly restructuring cellular DNA molecules. Rather than viewing genome evolution as a series of accidental modifications, we can now study it as a complex biological process of active self-modification.

## 1. Introduction

The title of this mini-review is a paraphrase of Dobzhansky’s famous dictum, “Nothing in biology makes sense except in the light of evolution” [1,2]. The reason for this paraphrase is to emphasize how, since Dobzhansky’s day, genetics and evolution science have moved into the era of a revolutionary new technology, DNA sequencing. Whenever such a technological revolution occurs, science must always ask itself what impact data from the new methods has on the validity of prevailing concepts.

Our current ideas of heredity center on DNA replication and transmission [3]. Although cell and organismal heredity involves transmission of all cellular molecules, we conventionally view RNAs, proteins, lipids, polysaccharides and other biomolecules as derivative products resulting from biochemical activities determined by genomic DNA sequences [4]. According to this DNA-centered perspective, stable inherited changes in organismal properties result primarily from alterations in the genome. New DNA sequences can encode new biochemical capabilities that lead to novel traits. If evolution is the acquisition of new characters over time [5], the most basic causal events should emerge from the processes that generate new DNA sequences. Those events are traceable in genome sequences, which today serve as the ultimate empirical data to test evolutionary hypotheses.

In this brief review, we will examine some of the evolutionary lessons from genome sequence data. Given the broad extent of the subject matter, only a select range of examples will receive attention. The investigation shows that many evolutionary changes result from cellular processes that produce abrupt changes in genome structure and organismal characters. These processes include symbiogenesis, horizontal DNA transfer, hybrid speciation and natural genetic engineering, especially the action of mobile DNA elements [6,7]. At the molecular level, evolution is often saltational rather than gradual [8], and punctuated equilibrium [9] is the default pattern to expect in the history of phenotypic properties. As we shall see, this view is quite different from Dobzhansky’s thinking. In particular, molecular sequence analysis documents many genomic elements and novel forms of hereditary variation that were hardly conceivable in Dobzhansky’s lifetime.

## 2. The Genome as a Highly Formatted Sequence Database

Genomes serve as controlled read-write databases for the reliable transmission and regulated expression of DNA sequence information [7,8,10]. Because controls over expression, replication and transmission are essential to survival and reproduction, genomes contain abundant *cis*-acting DNA formatting elements in addition to coding sequences determining RNA and protein primary structures [11].

Many of the formatting DNA elements are generic and present at multiple genome locations. Consequently, repetitive DNA is a major component of all genomes, and repeats frequently represent the majority of an organism’s DNA [12]. In the human genome, the repetitive content is about two-thirds of the total DNA, compared to well under 5% for protein-coding sequences [13].

For evolution, the significance of genome formatting by repeat DNA elements is that important phenotypic alterations can occur as a result of changes to so-called “non-coding” sequences. Recent discoveries about the functions of “non-coding” ncRNA molecules illustrate this point [14,15,16,17,18,19], as do Evo-Devo findings that many developmental alterations distinguishing related organisms occur in regulatory DNA elements rather than coding sequences [20,21].

## 3. Molecular Phylogenies Based on Core Information-Processing Systems

The most basic impact of genomics on evolution science has been the use of sequence data to establish relationships among different organisms. In the 1970s, Carl Woese pioneered molecular phylogenetic methodologies [22]. Woese chose to base his initial phylogenies on the sequence of small subunit ribosomal RNA for two reasons: (i) ssrRNA was abundant and amenable to 1970s sequence analysis methods; and (ii) the ribosome is a highly conserved central component of information transfer from the genome to the proteome. Because all cells have similar but still distinctive ribosomes, this organelle provided phylogenetic data to establish connections between very diverse organisms.

Using ssrRNA sequence analysis, Woese and his colleagues unexpectedly discovered that there are in fact two separate kingdoms of prokaryotic organisms (those lacking separate cell nuclei), not one bacterial kingdom as previously believed [23]. The two groups of prokaryotic organisms turned out to exhibit ssrRNA sequence clusters as phylogenetically distant from each other as both clusters were from the ssrRNA sequences of eukaryotic (nucleated) organisms. The evolutionary distance between the two prokaryotic groups was further confirmed by major differences in their cell membranes as well as by differences in the basic processes of genome replication and expression. The newly discovered prokaryotic cell kingdom was labeled Archaea because the first members to be studied were organisms isolated from extreme environments thought to resemble the early Earth [24]. Today, however, there is no reason to assume that Archaea are any more ancient than Bacteria.

The most basic tenet of evolution science—the genetic relatedness of all living organisms—is abundantly supported by molecular biology, in particular the universal features of the triplet code for amino acids and the similarities of core cell structures, like the ribosome, associated translation factors [25], and DNA and RNA polymerases. Nonetheless, the first phylogenetic application of genomics revealed a previously unknown complexity in the biosphere and, as we shall see in the next section, provided compelling evidence for a large number of evolutionary processes excluded by conventional evolutionary thinking based on population genetics and the Modern Synthesis [26,27]. As highlighted below, the phylogenies of Bacteria, Archea, eukaryotes and giant viruses continue to pose intriguing challenges in understanding the networked evolutionary connections between all domains of life [28].

## 4. Eukaryotic Origins and Major Eukaryotic Taxonomic Originations through Symbiogenesis

The identification of an unsuspected bifurcation among prokaryotes, the most abundant living organisms [29], immediately raised questions about the historical relationships between Archaea and Bacteria without nuclei, on the one hand, and Eukarya, the organisms with nucleated cells, on the other. Eukaryotes formed a coherent separate group based on ribosomal RNA sequences, and molecular phylogenies of different eukaryotic groups confirmed well-established taxonomic classifications, such as fungi, plants and animals. But the generic eukaryotic cell was closer to Bacteria in some features—membrane composition, metabolic pathways—and closer to Archaea in other features—replication, transcription and translation [30,31]. This phenotypic dichotomy gave support for longstanding but hotly disputed arguments championed by Lynn Margulis and others that symbiogenetic cell fusions served to create complex eukaryotic cells from simpler prokaryotic progenitors [29,32,33,34,35,36].

### 4.1. Endosymbiotic Bacterial Origins of Eukaryotic Organelles

Molecular phylogenetics unequivocally documented the role of symbiogenesis in Eukarya evolution by analysis of the two cell organelles that have their own genomes: (1) the mitochondrion, carrying out oxidative metabolism; and (2) the chloroplast, carrying out oxidative photosynthesis. All eukaryotic cells contain a functional mitochondrion or a non-oxidative derivative organelle [37,38,39]. Thus, the ancestral eukaryote must have acquired an endosymbiotic mitochondrial precursor, and molecular phylogenetics unambiguously identified the mitochondrion as belonging to the Alpha-proteobacteria group [40,41,42]. Similarly, photosynthetic eukaryotes, including red and green algae and plants, have chloroplasts, and molecular phylogenetics identified the chloroplast as a member of the cyanobacteria [43,44,45]. The chloroplast, and derivative plastid organelles in phylogenetically related non-photosynthetic eukaryotes, therefore must have descended from one or more endosymbiotic cyanobacteria [46,47].

### 4.2. DNA Transfer between Endosymbiotic Organelle and Nuclear Genomes

The genomes of mitochondria and chloroplasts do not encode all the proteins inherited from their bacterial ancestors. Many organelle proteins are encoded in the nuclear genome as a result of DNA transfers into the nuclear genome from mitochondria [48,49,50,51] and from chloroplasts [52,53,54,55]. These intracellular DNA transfers from one genomic compartment to another continue and have been analyzed in real time [56,57,58,59,60]. Interestingly, mitochondrial DNA transfer to the nucleus affects life-span in yeast cells [61], increases with age in rat cells [62], generates inherited disease loci in humans [56,63], occurs frequently in cancer cells [64], and participates in nuclear double-strand DNA break repair in organisms as distant as yeast and humans [56,65]. Chloroplast to nucleus DNA transfer is stimulated by stress conditions [59]. There is further evidence of organelle genomes acquiring nuclear and other external DNA [66,67,68]. Active import of DNA into plant mitochondria has been documented in real time [69].

Since the genetic content of mitochondria, chloroplasts and plastids differs significantly among taxonomic groups, important factors in eukaryotic taxonomic divergence are organelle to nucleus DNA transfer [49,50,70] and organelle genome restructuring: in mitochondria [51,71,72,73,74,75,76,77,78,79,80,81,82,83,84,85,86,87], by loss of mitochondrial oxidative capacity [38,88,89,90], in chloroplasts [45,47,54,91,92,93,94,95,96], and by loss of chloroplast photosynthetic capacity [47,54,97,98,99,100].

### 4.3. Origins of Photosynthetic Eukaryotic Taxa by Secondary Endosymbiogenesis

A large number of photosynthetic eukaryotes did not evolve directly from algae or plants and are most closely related taxonomically to non-photosynthetic organisms [37]. These organisms originated from “secondary” eukaryote to eukaryote symbiogenetic events where a red or green alga has become an endosymbiont in the initially non-photosynthetic lineage [101,102,103,104,105,106]. The resulting photosynthetic cells have four different genome compartments that exchange DNA segments: nucleus, mitochondrion, plastid and nucleomorph (descended from the algal nucleus) [107,108,109,110,111,112,113]. As with mitochondria and chloroplasts, the major intracellular DNA transfers occur from the organelles, including the nucleomorph, into the nuclear genome. The photosynthetic taxa arising from secondary endosymbiosis include euglenids and chlorachniophytes from green algal endosymbiosis and the chromalveolates from red algal endosymbiosis [37]. The large chromalveolate phylum includes major photosynthetic organisms responsible for a large fraction of atmospheric oxygen, such as brown algae, dinoflagellates and diatoms.

Photosynthetic endosymbiosis is not restricted to unicellular eukaryotes. There are cases where animals have acquired photosynthetic capabilities by symbiogenetic events [114,115,116]. The photosynthetic animals include sea slugs [117,118,119] and molluscs [120,121].

### 4.4. Formation of a Primitive Eye-Like Organ in a Unicellular Eukaryote by Serial Endosymbioses

Among the photosynthetic dinoflagellates resulting from algal ensymbiosis, there is a group labeled “ocelloids,” which possess a remarkable light-harvesting organ (the ocelloid) that resembles a complex camera-like animal eye [122,123]. The ocelloid has analogues to the cornea, lens, iris and retina. A noteworthy recent paper reports that genomic analysis reveals that each of these structures resulted from a distinct endosymbiogenetic event [124]:

“Here we show, using a combination of electron microscopy, tomography, isolated-organelle genomics, and single-cell genomics, that ocelloids are built from pre-existing organelles, including a cornea-like layer made of mitochondria and a retinal body made of anastomosing plastids. We find that the retinal body forms the central core of a network of peridinin-type plastids, which in dinoflagellates and their relatives originated through an ancient endosymbiosis with a red alga. As such, the ocelloid is a chimaeric structure, incorporating organelles with different endosymbiotic histories.”

The genomics-verified example of ocelloid formation by serial endosymbiogenesis has to be considered in light of Darwin’s famous statement: “If it could be demonstrated that any complex organ existed, which could not possibly have been formed by numerous, successive, slight modifications, my theory would absolutely break down.” [5] (p. 189). While the endosymbiogenetic origin of the ocelloid does not demonstrate the impossibility of camera eyes evolving by Darwinian gradualism, this striking case of adaptive evolution by repeated cell fusion events clearly lies outside the parameters of evolutionary processes envisioned by the author of *Origin of Species* as well as by the Modern Synthesis.

## 5. Eukaryotic Speciation by Inter-Specific Hybridization, Whole Genome Duplications and Genome Restructuring

When we ask how novel species arise from human intervention, it is significant that there are no cases where selection has led to species formation. Selection only modifies existing characteristics by reducing or amplifying them. Artificial species arise through hybridization, as in the case of the wheat-rye hybrid *Triticale* [125,126], and involve genome mergers and whole genome duplication (WGD) events [127,128]. A similar “cataclysmic evolution” process involving hybridization of wild grasses was at the origin of flour wheat (*Triticum*) several thousand years ago and can be reproduced in real time [129,130]. Ongoing abrupt hybrid speciation has been observed to occur in wild sunflowers [131]. A recent paper reports laboratory formation of a novel tobacco species with a double genome by fusion of tissue culture cells from two different natural *Nicotiana* species [132].

Many genomes contain duplicate copies of extended chromosome regions holding homologous genetic loci in a conserved (“syntenic”) order [133,134,135]. By documenting the prevalence of numerous syntenic duplications, genomic analysis provides compelling evidence that hybrid speciation and WGD have been common events in the history of evolution in all eukaryotic groups, including yeasts and other fungi [136,137,138,139], ciliated protists [140], plants [141,142] and animals, including two successive WGD events at the origins of vertebrates [143,144,145,146,147], followed by further WGD events in bony fishes [148,149].

In addition to WGD, interspecific hybridization leads to episodes of genome instability and restructuring [150,151,152]. Chromosome rearrangements have long been recognized as major features of speciation and taxonomic divergence [153,154,155]. Repetitive and mobile DNA elements play special roles in chromosome restructuring [156,157,158,159], as exemplified in primate evolution by the recently published gibbon genome [160].

## 6. Adaptations Acquired and Comingled by Horizontal Transfers

The conventional view of evolution maintained by Dobzhansky and his colleagues is that traits are transmitted vertically from progenitors to offspring, with evolutionarily important hereditary changes occurring within each particular line of descent. That is the pattern of “descent with modification” illustrated at the end of *Origin of Species* [5]. According to this perspective, each new adaptation has to evolve within a distinct lineage. In contrast to this conventional view, genomic analysis revealed that the molecular phylogenies of genetic loci encoding certain adaptive functions do not always match the taxonomic histories of the basal host genomes [30]. The genomic evidence indicated that organisms could acquire independently evolved DNA providing adaptive benefits from unrelated organisms by “horizontal” DNA transfer [161,162]. Genomic evidence shows that it often proves more efficient to adapt to a new ecological niche by borrowing functions from distant taxa rather than evolving them internally from the pre-existing genome.

### 6.1. Horizontal Transfer among Prokaryotes

The ability of prokaryotic organisms to exchange advantageous DNA segments is evident from studies of antibiotic resistance starting in the early years of molecular genetics (http://shapiro.bsd.uchicago.edu/ExtraRefs.AntibioticResistanceAndHorizontalTransfer.shtml) [163,164,165,166]. Horizontal DNA transfer can occur between prokaryotic cells by uptake of DNA released by cells to the environment (transformation), direct cell-to-cell contact (conjugation), or viral infection (transduction) [167]. Sometimes the horizontally acquired DNA constituted an independently replicating molecule in the prokaryotic cell [168], and sometimes the horizontally acquired DNA was integrated into the existing genome, often as extended “genomic islands” encoding many different proteins for complex traits like metabolism, defense and pathogenicity [169,170,171,172]. Special site-specific recombination structures called “integrons” exist in the genomes of some bacteria for the serial integration of antibiotic resistance cassettes [173,174] and, in particular species, “super-integrons” have accumulated cassettes encoding up to hundreds of diverse adaptive functions [175,176,177,178]. Horizontal transfer occurs across the Bacteria-Archaea divide and can even lead to the formation of novel taxa [179,180,181,182,183].

The prevalence of prokaryotic horizontal DNA exchange gave rise to the idea of a vast super-cellular pan-genome that prokaryotes can sample facultatively by transformation, conjugation and transduction to assemble novel genomes for cell adaptation to particular ecological niches [184,185,186]. This controversial notion has gained wider credibility in recent years as a result of metagenomic analysis, which has revealed unexpectedly large numbers of known and unknown coding functions present in environmental DNA samples, particularly those encapsidated in virus particles [187,188,189,190,191,192,193]. There has even been the suggestion that environmental metagenome data indicate the possible existence of major new cell types [194].

### 6.2. Horizontal DNA Transfer from Prokaryotes and Fungi to Multicellular Eukaryotes

Horizontal DNA transfer is not limited to prokaryotes. Genomic analysis provides abundant examples of eukaryotic adaptations with prokaryotic (and fungal) origins. These include:

- Biochemical pathways [195,196,197,198,199];
- Phytopathogenicity in *Botrytis* fungi [200];
- Ability to live in extreme environments [201];
- Capacity of plant parasitic nematodes to digest cellulose and other phytopolymers [202,203,204,205,206,207,208]. We know that this horizontal DNA transfer strategy was used repeatedly because each lineage of plant parasitic nematodes acquired their digestive enzymes from different fungi or bacteria;
- Energy metabolism and defense functions subject to purifying selection in a marine shrimp [209];
- Sequences of unknown but selectively conserved function transferred from marine bacteria to fish after the divergence of teleosts from other vertebrates [210].

Many bacteria live as endosymbionts in animals, and there is abundant evidence that parts or all of endosymbiont genomes have be incorporated into host genomes [211,212,213,214,215]. In *Drosophila* *ananassae*, for example, more than 2% of the genome comes from *Wolbachia* endosymbionts [216].

### 6.3. Horizontal Transfer from Eukaryotes to Bacteria

Although less widely documented than prokaryote to eukaryote horizontal transfer, the analysis of endosymbiotic and pathogenic bacteria infecting eukaryotic cells has turned up examples where these prokaryotes appear to have integrated eukaryotic host cell sequences into their genomes [217,218,219,220,221]. The restricted taxonomic distribution of the eukaryotic domains among the infectious bacteria indicates recent horizontal acquisition rather than shared vertical ancestry with eukaryotes [217].

Bacteria use the proteins containing typically eukaryotic domains encoded by horizontally acquired DNA sequences as injected “effector” molecules to modulate host cell metabolism and defenses in order to facilitate the infection process [222,223,224,225,226,227,228,229]. The ability of many infectious bacteria to grow in diverse eukaryotic hosts, such as amoebae and mammals [230], apparently plays an important role in the acquisition of eukaryotic domains from one kind of host (e.g., amoebae) and their utilization as invasion functions in another kind of host (e.g., mammals).

### 6.4. Horizontal DNA Transfer among Eukaryotes

In addition to fungi, other eukaryotes can transfer DNA horizontally across taxonomic boundaries [162,231]. This phenomenon is prevalent among unicellular protists [232,233,234], but various classes of DNA transfer have been documented between multicellular lineages:

- Mitochondrial genomes in flowering plants [235,236,237];
- Chloroplast genomes in plants [238];
- Mobile DNA elements [239,240,241,242,243,244,245];
- Sequences encoding diverse adaptive functions, including glyoxylate cycle enzymes in metazoa [246], photosynthetic carbon cycles in plants [247,248], anti-freeze proteins in fish [249], and mimicry pattern determinants in butterflies [250];
- Miscellaneous expressed functions acquired by a parasitic plant from its host [251].

Infectious bacterial endosymbionts and pathogens are widely considered as potential vectors for horizontal transfer between multicellular organisms [252,253,254,255,256]. Investigators have documented endosymbiont transfers between different multicellular host species [257,258,259], and many bacteria known as vertebrate pathogens also infect lower eukaryotes, especially amoebae [260,261,262,263,264,265,266]. Among these multivalent infectious bacteria, a number are capable of taking up DNA from their immediate environment [267,268,269,270].

### 6.5. Viral Integrations into Host Genomes

Viruses of all kinds (including RNA viruses) insert their genomes into eukaryotic host genomes with surprisingly high frequency [271,272,273,274,275,276,277,278,279,280,281,282,283,284,285,286,287,288]. Integration can occur by retroviral integrase functions, sometimes followed by recombination with other viral sequences [289], or by non-homologous end-joining (NHEJ) at DNA breaks [290,291]. Integration events at DNA breaks have the potential to generate novel sequence configurations.

### 6.6. The Amoeba-Megavirus “Melting Pot” of Sequences from All Three Cell Kingdoms

Viruses have long been considered both as substrates for evolutionary innovation [292,293] and as vectors for horizontal DNA transfer. Particular attention has recently focused on a group of Nucleocytoplasmic Large DNA Viruses (NCLDVs) with genomes comprising hundreds of thousands or millions of base-pairs [294,295,296]. Most significantly, NCLDVs acquire cellular genome fragments and have been found to carry a mixture of DNA sequences from all three major domains of life (http://shapiro.bsd.uchicago.edu/Viral_Composites.html) [297,298,299,300]. NCLDVs can infect both protists and multicellular hosts and thus transfer the incorporated cellular sequences. Amoebae are common hosts for many of these large DNA viruses, and amoeba have consequently been designated to constitute an evolutionary “melting pot” (http://shapiro.bsd.uchicago.edu/Amoebal_Viruses.html) [301]. The designation is especially appropriate for two reasons: (1) amoebae are phagocytic and can acquire DNA sequences from engulfed cells [302] and (2) amoebae are hosts to bacteria that both exchange DNA [303] and infect more complex eukaryotes, including both plants and animals [260,261,262,263,264,265,266].

Combining the phenomenology of viral infection, amoebael phagocytosis, multivalent bacterial infectivity and endosymbioses with the documentation of viral and prokaryote to eukaryote DNA transfers, it is more than clear that multiple pathways exist by which cells can acquire and transmit DNA segments from one eukaryotic host to another. Specific biochemical activities are distributed among distantly related domains of life [304,305], and we can expect further genomically-documented examples of horizontally acquired adaptations to multiply with continued genome sequencing.

## 7. Protein Evolution by Exon Shuffling and Exonization from “Non-Coding” DNA

Among the most striking results of genomics was the realization that many proteins and the DNA that encodes them are not continuous unitary structures. Many proteins consist of strings of functionally different but interacting “domains,” each one of which may be found iterated in distinct proteins [306,307,308]. Correspondingly, the cognate protein-coding regions are often discontinuous and composed of expressed coding segments (“exons”) separated by intervening segments (“introns”) [309,310]. While not always the case, DNA exons tended to encode functional protein domains or subdomains [311].

The segmented nature of proteins and protein-coding DNA has major implications for genome functionality and protein evolution:

- A given genetic locus can encode multiple protein products by joining different combinations of exons into the final mRNA by alternative splicing [312,313,314,315,316,317,318,319];
- New protein functionalities can arise rapidly by Lego-like assembly of exons from different sources, known as exon or domain shuffling [320,321,322,323];
- Totally new protein domains can originate rapidly by conversion of non-coding DNA segments into exons (“exonization”) and contribute to novel biochemical functions by subsequent duplication and exon shuffling [308,324,325,326].

Both the rapid evolution of new functionalities by exon shuffling and the origination of sequences encoding extended domains by exonization have potential for protein innovation beyond what is possible through codon-by-codon changes to existing proteins. Exon shuffling has an inherently high probability of producing adaptive novelties because it rearranges previously evolved sequences that encode established protein functionalities. This potential has been exploited in biotechnology where domain shuffling has proved an efficient method of protein engineering [327,328,329].

Mobile DNA elements play major roles in both exon shuffling and exonization. The genomic record indicates transposons and retrotransposons can incorporate and relocate exons [330,331,332,333,334,335,336,337], and mobile DNA-mediated exon shuffling has been studied in real time [338,339,340]. Genomics reveals numerous instances of exonization from mobile element insertions in humans and other mammals [308,326,341,342,343,344,345,346] as well as in plants [325].

## 8. Regulatory Signal Evolution Involving Mobile DNA Elements

The involvement of mobile DNA in genome change marks one of the most basic divergences between Dobzhansky’s Modern Synthesis perspective and a genomics-based view of the evolutionary process. The Modern Synthesis focused on isolated allelic changes at individual loci [347]. Mobile elements, on the other hand, are distributed at many sites throughout the genome and have the potential to generate coordinated changes rewiring distributed regulatory networks involving many loci (http://shapiro.bsd.uchicago.edu/Table5C-1.MobileElementsFoundtobeExaptedascis-RegulatoryControlSitesinAnimals.html) [8,348,349,350,351,352,353,354,355,356].

Genomics documents at least three episodes of regulatory innovation involving mobile elements in the course of vertebrate evolution [357,358], and mobile DNA has been a major source of regulatory motifs in human genome evolution [359,360]. Moreover, the fact that mobile elements are often the most taxonomically specific genome components potentially confers distinctive evolutionary trajectories on different lineages [361,362,363].

## 9. Adaptations and Innovations in Mammalian Reproduction Arising by Natural Genetic Engineering Processes Involving Mobile DNA and “Non-Coding” ncRNA Molecules

Because of their medical relevance, reproductive biology, embryonic development and stem cell biology in mammals have received particular attention from genomicists. The analysis has uncovered major roles for mobile DNA elements and ncRNAs derived from them. Rather than serving as “fossils that litter our genomes” [364], as conventional evolutionary thinking would assert, these elements are both essential evolutionary tools and active participants in contemporary genome function.

### 9.1. Retroviral Involvement in Placenta Evolution

Mammalian reproduction depends upon development of a syncytial placenta from the zygote to nourish the fetus during pregnancy. At repeated stages in mammalian evolution, distinct endogenous retroviral “envelope” (Env) proteins have been exapted as fusionogenic “syncytins” involved in forming placental tissue in different mammalian lineages (http://shapiro.bsd.uchicago.edu/Retroviral_involvement_in_placenta_evolution.html) [365,366,367,368,369,370]. Moreover, endogenous retroviruses (ERVs) provide transcriptional regulatory signals to direct imprinted expression of other proteins, such as insulin-like growth factor, required for placental function [371,372,373].

### 9.2. Mobile DNA Recruitment of Maternal Functions

The endometrium is the maternal tissue that nourishes the placenta in mammalian pregnancy. Endometrial development in the uterus involves the hormone-regulated expression of over 1,500 different proteins. The transcriptional regulatory signals coordinating biogenesis of this pregnancy-specific cohort evolved mainly from mobile DNA elements, both transposons and retrotransposons [374,375,376,377]. Convergent patterns of regulatory rewiring can be traced in the endometria of distinct mammalian lineages with well-sequenced genomes [378]. Thus, evolutionary innovations for both fetal and maternal sides of viviparous reproduction arose, to a large degree, through the ability of mammalian cells to mobilize repetitive components of their genomes to adaptive locations.

### 9.3. Mobile DNA and lncRNAs in Stem Cell Programming and Early Embryogenesis

Transcripts from human endogenous retroviruses (HERVs) have been found to be the most stage-specific RNAs expressed during early human embryonic development [379], and there is intrinsic retroviral reactivation in human pre-implantation embryos and pluripotent stem cells [19,380]. Although functional studies are not possible in human embryos, direct functionality has been established for a retroviral RNA in mouse, where MuERV-L transcripts expressed just 8–10 h after fertilization at the 2-cell stage are necessary for developmental competence at the 4-cell stage but not afterwards [381].

HERVs and other mobile DNA elements are the major components of long non-coding lncRNAs, which play important roles in (re)programming embryonic and stem cell genomes (“83% of lncRNAs contain at least one TE (transposable element), while of the total number of base pairs that comprise lncRNA sequences, 42% is derived from TEs” [382]). These include the lincRNA ROR (“regulator of reprogramming”) beginning with a HERV-H transcript needed for formation of human induced pluripotent stem cells (HiPSCs) [383,384], plus LINC01108 (Linc-ES3) and human L1TD1 lncRNAs required to maintain stem cell pluripotency [385,386]. A recent genomic census of over 4000 human-specific binding sites for the transcription factors which reprogram HiPSCs remarkably found between 99.8% and 100% of the sites to be located in mobile DNA repeats [387].

There is a burgeoning literature relating mobile DNA to the evolution of ncRNA-based circuitry essential for mammalian (and, more specifically, human) stem cell and embryonic development [351,375,384,385,388,389,390,391,392,393,394]. The lncRNAs bind to and tether a variety of epigenetic modification complexes that execute genome reprogramming [18,395], and the mobile DNA element sequences in each molecule have been proposed to constitute a combinatorial code of RNA domains that link together different genome modification processes (Called the “RIDL hypothesis” for Repeat Insertion Domains of LncRNAs) [394]. In addition to lncRNAs, mobile DNA elements have also been documented to be sources for cell regulatory miRNAs [396,397,398,399].

## 10. A 21st Century Evolutionary Principle: Cell-Mediated Variation of Read–Write (RW) Genomes

To recapitulate, a genomics-based view of evolutionary variation introduces novel features to hereditary control of cell biology impossible to predict when Dobzhansky and other evolutionary biologists formulated the neo-Darwinian Modern Synthesis in the middle of the last century:

- The existence of three distinct realms of cell evolution, Bacteria, Archaea and Eukarya;
- Symbiogenetic fusions involving these different realms leading to the formation of eukaryotic cells bearing organelles with multiple genome compartments;
- Horizontal organelle, virus and DNA transfers affecting adaptive traits across all cell types;
- The functional organization of proteins as systems of distinct interacting domains encoded by exons and subject to rapid evolution by exon shuffling and exon origination from non-coding DNA (exonization);
- Establishment of adaptive, distributed genome networks integrated by mobile DNA elements dispersing repetitive regulatory signals to multiple loci;
- Regulation of cell differentiation in multicellular development by non-coding lncRNA molecules composed largely of mobile repetitive DNA elements that serve as scaffolds for epigenetic modifying activities.

Altogether, the combinatorial coding and regulatory aspects of cell heredity, plus the biochemical abilities cells possess to rearrange DNA molecules, constitute a powerful toolbox for adaptive genome rewriting. Revelations from genomic analysis oblige us to reconsider the simplifying assumptions made in the past two centuries about the nature of evolutionary variation. Rather than single gene traits, we recognize that all phenotypes involve coordinated activity by multiple interacting cell molecules. As summarized above, we have evidence that genomes contain abundant and functional repetitive components in addition to the unique coding sequences envisaged in the early days of molecular biology [12]. Instead of the “Constant Genome,” subject to accidental modification, we know today that cells possess “Read–Write Genomes” they can alter by numerous biochemical processes capable of rapidly restructuring cellular DNA molecules [6,8]. Genomics has modernized our understanding of the evolutionary process. Rather than viewing genome evolution as a happenstance series of copying errors, we are now in a position to study it as a complex biological process of active self-modification.
